# Studying evolution of the primary body axis in vivo and in vitro

**DOI:** 10.7554/eLife.69066

**Published:** 2021-08-31

**Authors:** Kerim Anlas, Vikas Trivedi

**Affiliations:** 1 EMBL Barcelona Barcelona Spain; 2 EMBL Heidelberg, Developmental Biology Heidelberg Germany; California Institute of Technology United States; California Institute of Technology United States

**Keywords:** stem cells, primary axis, evolution, developmental trajectories

## Abstract

The metazoan body plan is established during early embryogenesis via collective cell rearrangements and evolutionarily conserved gene networks, as part of a process commonly referred to as gastrulation. While substantial progress has been achieved in terms of characterizing the embryonic development of several model organisms, underlying principles of many early patterning processes nevertheless remain enigmatic. Despite the diversity of (pre-)gastrulating embryo and adult body shapes across the animal kingdom, the body axes, which are arguably the most fundamental features, generally remain identical between phyla. Recently there has been a renewed appreciation of ex vivo and in vitro embryo-like systems to model early embryonic patterning events. Here, we briefly review key examples and propose that similarities in morphogenesis and associated gene expression dynamics may reveal an evolutionarily conserved developmental mode as well as provide further insights into the role of external or extraembryonic cues in shaping the early embryo. In summary, we argue that embryo-like systems can be employed to inform previously uncharted aspects of animal body plan evolution as well as associated patterning rules.

## Introduction

Metazoans display vast morphological diversity, yet body plans (box definitions) can universally be distilled to the presence of one to three body axes. Contrasted with protists, a characteristic feature is the existence of a species-specific embryonic phase in the respective life cycle. During its early stages, often collectively termed as gastrulation, simultaneous rearrangement and differentiation transforms a collection of embryonic (stem) cells into a complex, multilayered structure consisting of two (diploblasts) or three (triploblasts) germ layers organized along at least one primary body axis.

Despite the conservation of gene regulatory networks that influence cell state and behavior, embryo geometry and overall tissue rearrangement dynamics can vary substantially during axial emergence (Kalinka and Tomancak, 2012). Nevertheless, the morphological outcome of this event – a multilayered body plan with its respective axes - remains constant throughout metazoans (Keller et al., 2003; Leptin, 2005; Solnica-Krezel, 2005; Solnica-Krezel and Sepich, 2012).

Traditionally, evolution of the primary axis has been studied from genomic and molecular perspectives in embryos across the animal kingdom, by examining the roles of, for instance, Wnt/β-catenin signaling and T-box genes that evolutionarily predate the cnidarian-bilaterian split (Sebé-Pedrós et al., 2013; Holstein, 2012; Loh et al., 2016). More recently, a plethora of in vitro model systems of early embryonic patterning have emerged which are also being scrutinized via transcriptomic, quantitative as well as functional assays (Gritti et al., 2021; Shahbazi et al., 2019; Moris et al., 2020b; Rossant and Tam, 2021; Rivron, 2021; Zernicka-Goetz, 2021). However, for the most part, such studies have focused on highlighting similarities to the respective source species. In turn, we here suggest that, through examination and broader comparisons of embryo-like in vitro or ex vivo systems with related entities and native embryos across animals, one may gain deeper insight into the emergence of metazoan body axes as well as the evolution of body plans.

Upon consideration of such systems from various species, including *Nematostella*, *Xenopus*, zebrafish, mouse and human, it transpires that isolated ensembles from embryonic stem cells (ESCs) and ESC-like populations (ESC-LPs, box definitions) universally harbor the capacity to self-organize (box definitions) into a rudimentary body plan with at least a primary axis. Notably, this may occur via developmental trajectories alternative to those in the respective native embryo. We hence argue that these observations could point toward a deeper, evolutionarily *conserved developmental mode* (box definitions) which cells exhibit as they are released from their species-specific geometrical arrangements and mechanochemical signaling environments (Newman and Bhat, 2008; Newman, 2016). Altogether, in this perspective, we outline metazoan body axes and conserved initial patterning genes, followed by a brief review and comparison of mostly recent embryo-like systems in an evolutionary context. Lastly, we speculate on the underlying mechanisms which may have led to the observed diversity in early embryo morphology and on shared, fundamental principles of initial body plan establishment across the animal kingdom.

### Animal body axes

Animals display a variety of body plans consisting of one or more anatomical axes that delineate body polarity and characterize the degree of symmetry in the arrangement of body parts around the axes. Notably, while the initial axis of the pre-gastrulating embryo is often labeled as animal-vegetal, based on the polarity of the oocyte, this may not necessarily correspond to the alignment of the body axes that generally emerge concomitantly with gastrulation or germ layer specification (Valentine, 2004; Willmore, 2012).

In cnidarians and ctenophores an oral-aboral (OA) axis is ubiquitously identifiable at least in larval stages. This further arguably includes placozoans, demarcated along their respective upper and lower epithelia, the latter of which is used for feeding and exhibits expression of oral-associated genes (DuBuc et al., 2019). Cnidarian polyps and medusae generally exhibit (bi-)radial or bilateral symmetry as opposed to the rotational symmetry of ctenophores (Dunn et al., 2015; Ball et al., 2004). Larval sponges feature an antero-posterior (AP) axis that is alternatively labeled animal-vegetal (AV) axis as well as radial symmetry. In contrast, adult sponges only display an apical-basal (AB) axis, due to clear absence of a morphological mouth and indistinct body symmetry (Swalla, 2006; Degnan et al., 2005; Ryan and Baxevanis, 2007; Leininger et al., 2014; Petersen and Reddien, 2009; Genikhovich and Technau, 2017).

As the second major group next to the diploblasts, all other extant animals are referred to as triploblasts or bilaterians. Despite there being considerable variability, the body plan of most bilaterian phyla features three distinct axes: Anteroposterior (AP), dorsoventral (DV), and mediolateral (ML, also labeled as the midline) as well as bilateral symmetry (Genikhovich and Technau, 2017).

Moreover, as previously alluded to, although adult body plans can be congruent with the axial emergence during earlier embryonic phases, this is not generalizable across metazoans: While the early gastrula of *Nematostella* exhibits radial symmetry around the OA axis, bilateral symmetry takes over at late gastrula stages (Genikhovich and Technau, 2017). This is further exemplified by echinoderms, where, in contrast to their larval form, adult starfish are radially symmetric with an OA axis (Ji et al., 2012).

Beyond the natural anatomical meaning and the morphological significance (feeding and digestion), studies have highlighted the convergent regulatory logic of AP and OA axis establishment via a Wnt/β-catenin-dependent system in deuterostomes and cnidarians (Kraus et al., 2016; Bagaeva et al., 2020). However, it has to be noted that, irrespective of the conservation of rudimentary gene expression domains and molecular mechanisms pertaining to initial specification, subsequent species- or phylum-specific patterning dynamics of these axes can vary substantially across the animal kingdom (Martindale and Hejnol, 2009). Therefore, it is crucial to consider the possibility of convergent evolution of anatomical features when evaluating the molecular signatures which demarcate them, as evident in case of dorsoventral patterning and trunk neuroanatomies across invertebrates (Martín-Durán et al., 2018).

For instance, in the context of bilaterian evolution, a direct correspondence between the cnidarian mouth and the bilaterian blastopore – a transient opening during the early gastrula stage of embryogenesis – is widely accepted. Yet, among bilaterians, there is substantial, species-specific secondary re-organization of mouth and anus formation (Martín-Durán et al., 2016).

Altogether, in spite of the scarcity of experimental data in case of ctenophores, placozoans and sponges, it is hence still reasonable to assume that the AP axis of bilaterians, the OA axis of cnidarians, ctenophores and placozoans as well as the AP or AV axis of sponges constitute the most evolutionary ancient and thus main or primary body axes in these organisms.

Apart from Wnt/β-catenin, transcription factor Bra/T is another well-studied marker of primary axis, and thus initial body plan patterning (Petersen and Reddien, 2009; Swalla, 2006). During gastrulation, Bra/T defines and patterns the blastopore in cnidarians and ctenophores, giving rise to the oral region, as well as the posterior and incipient mes(endo-)doderm in triploblasts (Swalla, 2006). In early *Sycon ciliatum* sponge embryos, elevated Bra/T transcriptional activity is localized to a region undergoing a characteristic inversion event which shapes the thus emerging larva (Leininger et al., 2014).

While generally associated with mesoderm formation in bilaterians, depending on the context and the species studied, it may be involved in specification of any of the three germ layers. What remains consistent, however, is the implication of Bra/T in cell movements or adhesion properties (Technau and Scholz, 2003), pointing toward its ancient role to regulate morphogenetic movements, such as invagination or folding, during gastrulation (Technau, 2001; Yamada et al., 2010).

Intriguingly, the emergence of animal multicellularity has been linked to Wnt signaling due to its implication as a facilitator of symmetry breaking (Loh et al., 2016) (box definitions) and having not been identified in unicellular eukaryotes, plants, or fungi (Holstein, 2012). Whereas in several studied species Bra/T has been found to act immediately downstream of the Wnt and the TGF-β pathways (Arnold et al., 2000; Vonica and Gumbiner, 2002; Turner et al., 2014; Latinkić et al., 1997; Pauklin and Vallier, 2015), T-box transcription factors themselves predate multicellularity, as they are present in genomes of several unicellular opisthokonts, including several fungi taxa as well as ichthyosporeans and filastereans (Sebé-Pedrós et al., 2013; Sebé-Pedrós and Ruiz-Trillo, 2017; Richter et al., 2018).

### Evolution of body plans in vivo and the significance of systems from ESCs and ESC-LPs

Despite the evolutionarily conserved role of Wnt and Bra/T in patterning of the primary body axis, the diversity of shape, size, dynamics of tissue rearrangements and the environmental niches across embryos of different species (Leptin, 2005; Solnica-Krezel and Sepich, 2012) points toward a vast space of functional developmental trajectories accessible to early embryonic cells. Furthermore, both within and between phyla there is evident topology shuffling within key developmental gene networks, especially in terms of co-option of novel or external genes into these regulatory modules, thereby creating novel developmental mechanisms via the cell-to-tissue-level morphogenetic processes they influence (Chen et al., 2013).

Such a perspective hints at a picture of evolution that transcends the simple conservation of genes to the conservation of dynamic developmental modes which unfold to generate similar axial coordinates across phyla, while the constituent cells themselves might take diverse developmental trajectories dictated by the embryonic and extraembryonic environment.

Understanding these developmental modes in vivo comes with the challenge that cells within the embryonic context are also constrained by distinct factors – embryonic (shape) and extraembryonic (signaling and mechanical cues) – that generate the observed morphological diversity. We note that such ‘constraints’ do not imply less sophisticated developmental processes, on the contrary, in many species, external input is strictly required for complex morphogenesis to occur (Sheng and Foley, 2012; Christodoulou et al., 2019).

Therefore, the study of ex vivo or in vitro systems can elucidate, on the one hand, shared principles and, on the other hand, provide an alternative angle: Removing stem cells (SCs) and SC-like populations from their native context potentially allows the disentanglement of such species-specific factors, thereby allowing cells to follow developmental trajectories guided by their inherent self-organizing capabilities (Sogabe et al., 2019).

This concept is well exemplified by the various kinds of embryo-like structures, termed embryoids or embryonic organoids that have been developed recently from either embryonic or induced pluripotent stem cells (iPSCs) to recapitulate patterning events of initial embryogenesis until the onset of organogenesis (Shahbazi et al., 2019; Moris et al., 2020b).

Box 1. Box definitions and abbreviations.

**Body plan** A set of morphological features describing the body shape and structure of a given species. The most fundamental aspects of body plans, such as body axes, are conserved even across phyla.

**ESC-LPs** Embryonic stem cell-like populations. We employ this term to summarize various kinds of cell populations which are capable of differentiating into the germ layers and assembling dynamically to generate a basic animal body plan. In this article, the term ESC-LP denotes pluripotent cells from different sources: induced (e.g. iPSCs) or present in vivo and spanning different states of the pluripotency spectrum (naïve versus primed ESCs). Such ESC-LPs are not specific to mammalian systems but can be present in species across the animal kingdom, including diploblasts and arthropods.

**Self-organization** Emergence of order based on local interactions between different parts of a system that was initially disordered. Characteristic of such a process is feedback between components that amplifies the effects of local interactions or disturbance to the global level. In the context of aggregation experiments of ESCs and ESC-LPs discussed in this review, emergence of the primary axis can be considered self-organization. A related concept in biological systems is that of *genetically encoded self-assembly* (Turner et al., 2016), meant to imply processes where the information for the emergence of order is already encoded in the constituent cells that are primed to form structures or patterns under the influence of genetic programs.

**Conserved developmental mode** Cell ensembles from ESCs grown in absence of species-specific external (micro-)environments and cues adopt a set of differentiation trajectories in terms of both morphogenesis and gene expression which are evolutionarily conserved across different species, in order to form patterned structures resembling actual embryos (**Figure 3**). A similar term, *dynamical patterning* or *morphogenetic module* (DPMs) refers to evolutionary conserved sets of gene products as well as their physical effects that alter the state, shape, size and arrangement of cells in a given population (Newman and Bhat, 2008; Newman, 2016). Therefore, several of such DPMs can be activated as ESCs or ESC-LPs revert to the conserved developmental mode.

**Symmetry breaking** Emergence of an asymmetry, morphological or molecular, i.e. in terms of gene expression, within a previously homogenous structure. Here, we refer to symmetry breaking in the context of asymmetries at the level of cell populations rather than asymmetries displayed by single cells of an aggregate.

Historically and perhaps conceptually, they are preceded by SC embryonic explant and re-aggregation systems (Wilson, 1907), several of which were established already decades ago in evolutionarily distant species such as *Hydra* and starfish (Wilson, 1911; Gierer and Meinhardt, 1972; Dan-Sohkawa et al., 1986). These and related research efforts have underscored the regenerative capacity as well as the self-organizing potential of ESC-LPs across metazoans, given that, in both species, and thus across clades, re-aggregated cells are able to reconstitute a functional animal. Concomitantly with the emergence of new in vitro embryo-like structures, a few novel examples of such ex vivo systems have also been reported as of late.

In what follows, we will list mostly recent examples from both diploblasts and triploblasts and discuss selected ones as to how far these can provide clues for elucidating universal principles of animal body axis specification. While we aim to present an evolutionary perspective with as much phylum coverage as possible, it has to be noted that relevant data on non-mammalian systems is rare and limited to re-aggregation approaches.

### Re-aggregation studies in cnidarians

In the cnidarian model organism *Hydra*, single-cell dissociation and reaggregation experiments were already performed decades ago and yielded insights into regeneration and tissue developmental processes (Wilson, 1911; Gierer and Meinhardt, 1972). Remarkably, *Hydra* exhibits vast regenerative potential with any extracted fragment of its body being able to reconstitute an animal (Vogg et al., 2019). Labeling experiments have demonstrated that pattern formation in re-aggregates arises de novo and that cells sort themselves only in regards to their germ layer identity (Technau and Holstein, 1992; Technau et al., 2000).

A more recent study analyzes the dissociation and re-aggregation of *Nematostella* mid-gastrulae in order to elucidate developmental plasticity of embryonic cell populations (Kirillova et al., 2018). Oral halves, which contain a population of blastopore lip cells with axial organizer capability conveyed by Wnt1 and Wnt3, were able to reorganize the body plan and develop into a functional polyp. Accordingly, aboral halves were only capable of reconstituting an animal upon prior injection of both Wnts, demonstrating that a competent cell population (ESC-LPs) is needed for generation of a re-aggregate capable of developing into a functional animal.

Furthermore, these experiments suggest that this competence can be retroactively conveyed by addition of relevant factors into cells which have previously undergone a separate differentiation trajectory, similar to the reprogramming strategies used for induced pluripotent stem cells (Takahashi and Yamanaka, 2006). Therefore, a stem cell population in a more naïve state, from embryos prior to the gastrula stage, should, in theory, harbor the full developmental potential to self-organize the body axes without external inputs.

Akin to corresponding experiments in adult *Hydra*, cells in developing embryonic *Nematostella* re-aggregates exhibit vast reprogramming of their axial identity. Notably, while ectodermal cells could convert into endoderm, the latter remained endodermal. However, in case re-aggregates were generated purely from endoderm, cells became mesenchymal and migratory causing aggregates to collapse.

Perhaps, most intriguing though is the observation that re-aggregates which successfully reform a functional animal employ a developmental mode distinct to normal *Nematostella* embryogenesis, but similar to other cnidarians, such as hydrozoans. Namely, instead of invagination, germ layer specification occurs via delamination of the ectoderm, ingression of those endodermal plate cells that were initially located at the aggregate surface due to random mixing, as well as cavitation of inner cells.

### Explants in anamniotes

The field of amphibian (and avian) development has had a long history of excising tissues from the native embryo and either transplanting them in a different embryo or different regions of the same embryo or culturing them as ex vivo explants over extended periods (Mookerjee, 1953; Ball, 1966; Hamburger, 1969; Frederick, 1991). While these studies have provided crucial insights into the developmental potential of cells through the comparison of ex vivo explants from different stages of development (Keller and Danilchik, 1988), a detailed picture of the self-organizing capabilities of cells, akin to reaggregation studies in cnidarians or 3D aggregates of ESCs from mammals, is emerging only recently.

In *Xenopus*, cell aggregates obtained by dissociating chordamesoderm (prospective notochord) from the early gastrula were shown to undergo cell sorting and convergent extension to establish an anteroposterior patterning in an elongated structure revealed through the expression of Bra/T (Xbra) and Chordin (Ninomiya et al., 2004). A more drastic example is that of aggregates of cells from dissociated animal cap explants at an earlier developmental stage. By addition of Activin A to these cells, they were shown to generate multiple mesodermal tissue types (Green and Smith, 1990) and, depending on the dosage of the signal, these consist almost entirely of notochord (Green et al., 1992). Perhaps, even more remarkable is the scenario when such dissociated cells are allowed to re-aggregate spontaneously: They round up into a ball followed by elongation and organization of an axis in a manner whereby the axial extrusion resembles archenteron elongation in deuterostomes, for instance sea urchins (Green et al., 2004).

Similar to amphibians, different types of explants from teleost embryos have also been used historically to explore the self-organization of cells. It was shown that isolated blastoderms of *Fundulus heteroclitus*, deprived of yolk, can develop into embryo-like structures (Oppenheimer, 1936). Furthermore, uncommitted embryonic cells of animal caps in 128-cell stage zebrafish embryos were shown to organize a complete embryonic axis upon injection of opposing gradients of BMP and Nodal (Xu et al., 2014).

Recently an ex vivo system, termed pescoids (Trivedi et al., 2019) in analogy to mammalian gastruloids, utilized most of the blastodermal cells severed from yolk in zebrafish embryos around 256 cells stage, before germ layer induction. These ex vivo systems, when allowed to grow without the supply of external signals, were shown to be robust to cell mixing, able to form a polarized domain of high Bra/T (tbxta) expression, leading to further elongation under the influence of Nodal (similar to amphibian requirement for Activin) and non-canonical Wnt (PCP) signaling. In addition, they specify all germ layers and most mesodermal lineage precursors in terms of marker gene expression (Fulton et al., 2020; Schauer et al., 2020; Williams and Solnica-Krezel, 2020).

Lately, such explant system have also been derived from the Mexican tetra *Astyanax mexicanus* (Torres-Paz and Retaux, 2021) and the Japanese rice fish or medaka *Oryzias latipes* (Zilova et al., 2021). In the latter case, blastoderm cells were re-aggregated and exposed to factors which facilitate neural development to generate anterior structures recapitulating retinal differentiation as well as morphogenesis.

Results in amniotic embryos need to be interpreted with care since the embryonic cells inherit maternal signals starting at the single cell stage, as it was indeed shown for pescoids that require polarized inheritance of maternal factors (Schauer et al., 2020). A definite demonstration of spontaneous self-organization requires availability of ESC-LPs for teleosts and efforts toward culturing ESCs for medaka will prove to be a valuable tool in this regard (Hong et al., 1996).

In their natural settings, the phenomenon of complete dispersion of pre-embryonic blastomeres in annual killifish (*Austrofundulus myersi* Wourms, 1972, *Austrolebias charrua* Pereiro et al., 2017), followed by their re-aggregation to generate embryonic body plan is the closest observation to complete dissociation of teleost cells. While it is not clear to what extent the organization of dissociated cells is autonomous and independent of extraembryomic cues, annual killifish, intriguingly, represent an exceptional in vivo testimony to the notion of multiple developmental trajectories being available to the embryonic cells as seen in the differential rate of specification of anterior and posterior structures in embryos that have and have not undergone diapause (Podrabsky et al., 2010; Romney et al., 2018).

### Mammalian in vitro systems

In the past few years, a diverse array of mammalian in vitro embryonic models has emerged, generated from either mouse or human ESCs (Shahbazi et al., 2019; Baillie-Benson et al., 2020; Ghimire et al., 2021; Gupta et al., 2021). Pioneering work on 3D aggregates, termed embryoid bodies (EBs), has demonstrated that these can give rise to progenitor cells for the germ layers as well as form rudiments of tissues and organs without the context of an embryo (Doetschman et al., 1985; Desbaillets et al., 2000; Brickman and Serup, 2017). Polarized gene expression and self-organized axial emergence in embryoid bodies has been shown to be mediated by Wnt signaling (ten Berge et al., 2008; Sagy et al., 2019), although the extent of displayed axial organization is limited.

#### Mouse gastruloids

mGastruloids are initially spherical aggregates derived from few hundreds of homogenous mESCs which, although removed from extraembryonic tissues and nearly all associated signaling and mechanical cues, mimic some morphogenetic events of early mouse embryos, such as elongation, germ layer as well as axis formation and associated patterning (van den Brink et al., 2014; Beccari et al., 2018). Initially mGastruloids were developed motivated by the observation that, under differentiation conditions, mouse embryo P19 carcinoma cells are able to form polarized, elongated structures (Marikawa et al., 2009). Lately, they have also been extrapolated to hESCs (hGastruloids) (Moris et al., 2020a; Marikawa et al., 2020).

AP axis specification in aggregates is demarcated by autonomous polarization of previously homogeneously distributed canonical Wnt and Bra/T expression, constituting the first system-wide symmetry breaking event identified thus far (Turner et al., 2017). After 6–7 days in non-adherent culture, mGastruloids not only develop transcriptionally demarcated AP, DV, and ML axes, but also display spatiotemporal expression of hox gene clusters similar to those in mouse at roughly embryonic day (E) 9.0–9.5 (Beccari et al., 2018).

#### Human gastruloids

Recently established hGastruloids constitute a model for human early AP patterning (Moris et al., 2020a). Morphologically resembling their mouse counterpart, they are generated from few hundreds of hESCs, pre-treated with a WNT agonist for 24 hr in 2D culture, then aggregated in low adherence, differentiation conditions with ongoing WNT upregulation that is subsequently diluted. BRA/T develops polarized expression a day after aggregation, thereby demarcating the posterior pole which subsequently elongates.

Already after 3–4 days of development, hGastruloids reach maximal elongation and exhibit expression of genes associated with all three germ layers as well as a plethora of AP axial patterning genes, spatially organized in a manner similar to mammalian embryos. These notably include anterior cardiac mesoderm- and posterior node-related genes as well as a posterior to anterior somitogenesis signature. Hence, in terms of their transcriptional profile, hGastruloids partly correspond to CS9 human embryos. Akin to mGastruloids, for the most part, they do not seem to recapitulate early human-specific embryonic morphology.

### Common developmental trajectories accessible by ESC(-LP)s across species

**Figure 1.**
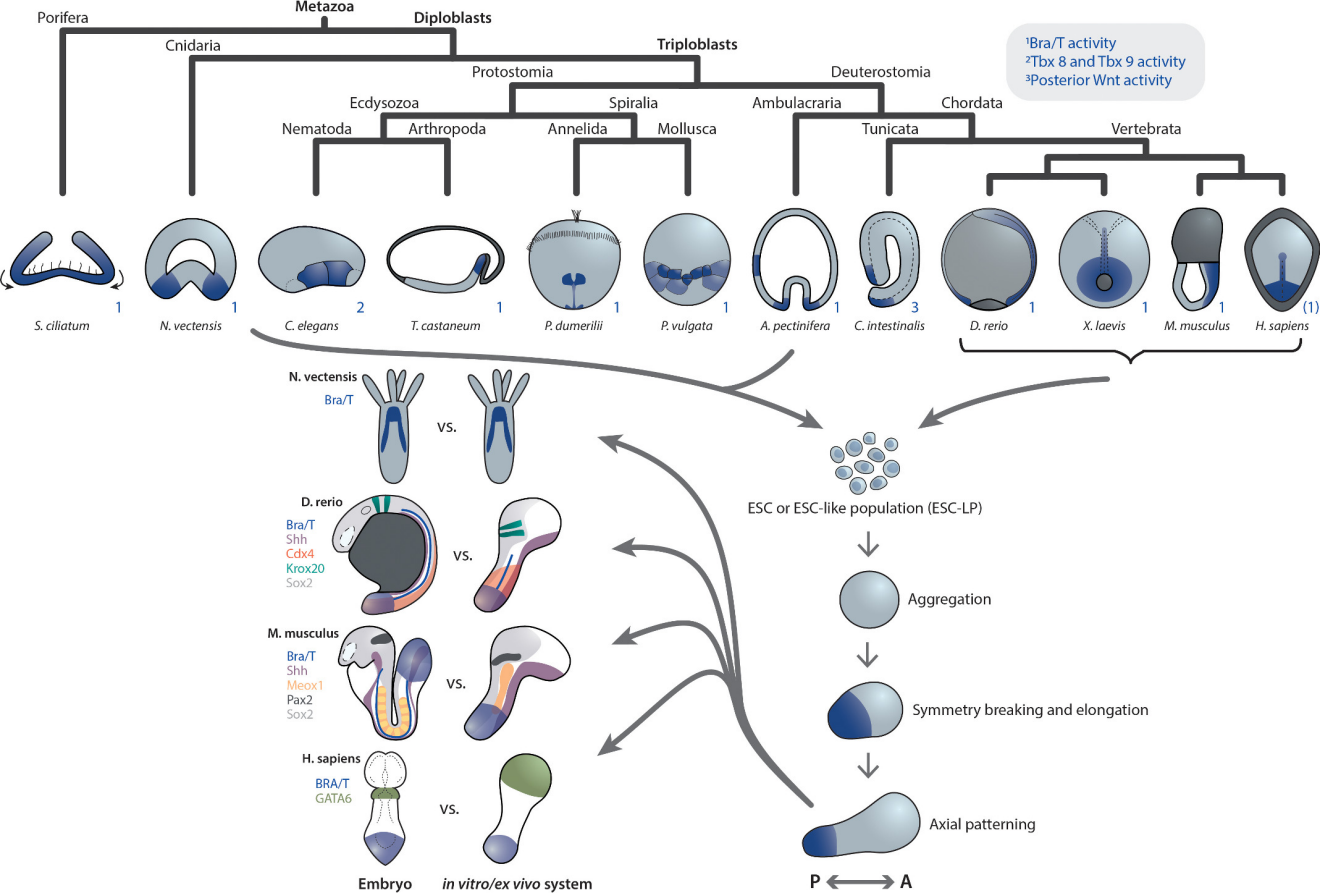
Primary axis formation during gastrulation in metazoan embryos and corresponding artificial systems. Metazoan embryos around the gastrulation or an equivalent developmental phase exhibit distinct morphologies and associated overall tissue rearrangement dynamics. Yet, during this event they universally specify at least a primary body axis, demarcated by conserved expression of posterior (or oral in cnidarians) patterning determinants. Among them are T-box and Wnt genes, localized transcriptional activity of which is highlighted in blue. Note that marker gene expression patterns in the human embryo are speculative. Research efforts have shown that ESC or ESC-like populations from species across the animal kingdom can be (re-)aggregated in vitro and, although lacking the respective external environment and associated developmental cues, remain capable of recapitulating at least a basic transcriptional body plan with an anteroposterior (AP) and oral-aboral (OA) axis, respectively. Strikingly, comparison of examples of such in vivo or ex vivo systems highlights a remarkable overall similarity despite the varying geometry of the respective native embryo. This may point toward the existence of a conserved developmental mode that cells exhibit when released from their species-specific extraembryonic environment.

Despite the differences in early embryogenesis of source species, studies involving aggregates of corresponding ESC(-LPs) in *Nematostella*, zebrafish, *Xenopus*, mouse, and human embryonic model systems, reveal several unifying observations as well as interpretations. Particularly with respect to the establishment of the primary axis, cell aggregates develop an oral or posterior domain characterized by Bra/T expression in the native embryo, demarcating a symmetry-breaking event (Figure 1), followed by axial elongation and AP patterning.

In addition, a morphological similarity – initially spherical, then elongated – can be observed between the different systems, notwithstanding the strikingly distinct geometries of the *Nematostella*, fish, mouse and human embryos. While the precise mechanism remains to be elucidated, another commonality is the analogous dependence of primary axis formation on the initial number of cells in aggregates from two evolutionarily highly distant species: Larger re-aggregates from *Nematostella* gastrulae (Kirillova et al., 2018) as well as mouse ESCs (gastruloids van den Brink et al., 2014 and embryoid bodies ten Berge et al., 2008) form multiple oral and posterior domains, respectively.

Altogether, aforementioned developmental similarities seem to reflect an inherent, converging self-organizing capability of ESCs and ESC-LPs across species when developing in the most minimal environment with the least possible external biochemical and mechanical input.

We argue that this capability represents an evolutionarily conserved developmental mode which we define as the set of developmental trajectories – in terms of morphogenesis and gene expression dynamics – that cell collectives within a given system or aggregate from ESCs undergo as they give rise to a patterned, embryo-like structure in absence of species-specific external (micro-)environments and associated cues.

**Figure 2.**
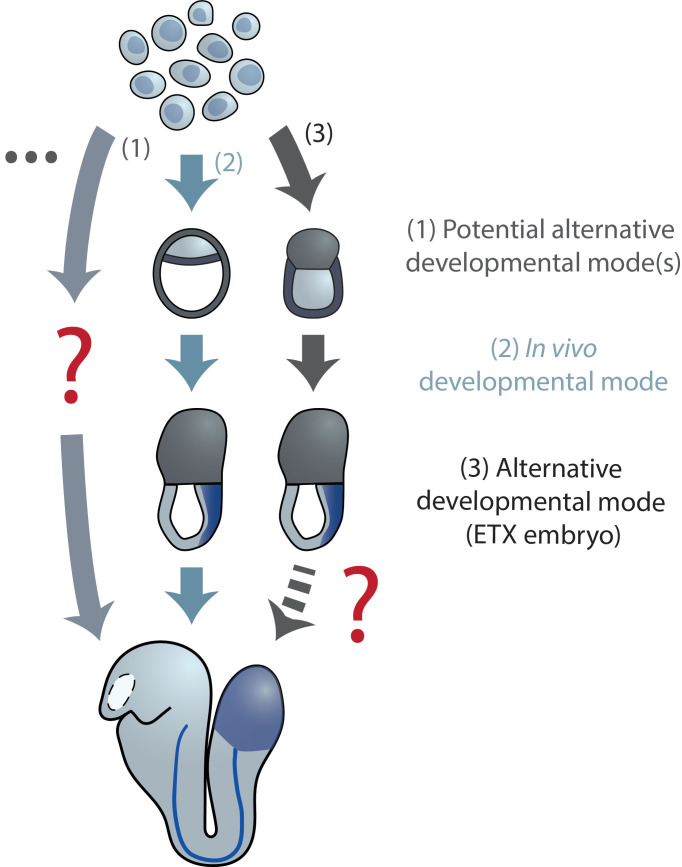
Diverse developmental trajectories accessible to ESC(-LP)s in the presence and absence of extraembryonic inputs. Compared to the native embryo, (re-) aggregation of ESC-LPs reveals potentially alternative developmental modes to the same body plan. Evidence for this has been found in Nematostella reaggregates and mGastruloids (Kirillova et al., 2018; Hashmi et al., 2020; Vianello and Lutolf, 2020). (i)ETX embryos (Sozen et al., 2018; Zhang et al., 2019; Amadei et al., 2021) from mESCs, TSCs and XEN cells could point toward the existence of such an alternative mode, as they form structures resembling the actual gastrulating mouse embryo without closely mimicking blastocyst morphology (stages E3.5–4.5) earlier during their development.

It is noteworthy that this can indeed differ from the mode employed by the native embryo that uses a subset of (morphogenetic) trajectories available to the highly developmentally plastic embryonic cells (Figure 2). This plasticity is, for instance, exemplified by re-aggregates from *Nematostella*, in which, during germ layer specification, trajectories of cells resemble those of cnidarians, such as hydrozoans. Yet, the end result is a functional animal, alike to one formed through normal *Nematostella* embryogenesis (Kirillova et al., 2018). In case of mGastruloids, cells expressing endodermal markers appear to be specified in a manner distinct to the embryo, as they arise dispersed and subsequently congregate without the requirement of an epithelial-to-mesenchymal transition (EMT) (Vianello and Lutolf, 2020; Hashmi et al., 2020).

Moreover, it was recently shown, through a transcriptional comparison of early mGastruloids to the mouse embryo, that while the emerging mesendodermal lineages in vitro are similar to their in vivo equivalents, AP axis and concomitant germ layer specification occurs in a parallel, but distinct manner, initiated by a more mesenchymal-like primed ESC population as opposed to the epithelial epiblast cells of the native embryo (Anlas et al., 2021).

Complementary to the idea of ESC(-LP)s unraveling a conserved developmental mode under minimal culture conditions, is the observation that cells on a separate, advanced differentiation path can be coaxed to follow such basal trajectories when presented with the right factors. In re-aggregates from *Nematostella* aboral halves, prior injection of Wnts is required to prompt the cells to develop into a functional animal (Kirillova et al., 2018). Conversely, in gastruloids from induced pluripotent stem cell (iPSC) lines, AP patterning dynamics, including initial Bra/T polarization, are similar to their ESC counterparts (Beccari et al., 2018).

### Significance of extraembryonic inputs for recapitulating embryo-like patterning

Embryo-like structures also serve as a valuable tool to study the divergence of developmental trajectories in a systematic manner by exposing ESC(-LP)s to species-specific cues. These can be applied in manifold ways, for instance via adding early extraembryonic cells such as TSCs (trophoblast stem cells) or XEN (extraembryonic endoderm stem-) cells which in mammals give rise to tissues that closely interact with and instruct embryo patterning (Arnold and Robertson, 2009; Langdon and Mullins, 2011). Further options comprise the timed and localized application of signaling molecules to mirror endogenous inputs and the use of ECM mimics, such as hydro- or matrigel (Hughes et al., 2010; Caliari and Burdick, 2016) to generate 2D or 3D scaffolds and hence cues through mechanochemical interactions.

Indeed, several recently described systems which implement such principles are allowing us to dive deeper into the question of how embryonic cells can generate a richer diversity of cell states and embryo-like morphology in the presence of aforementioned external cues.

### Mouse - ETS and ETX embryos

Single mESCs and TSCs, when grown together in a 3D Matrigel scaffold, mutually cooperate to assemble into structures, termed ETS embryos, highly reminiscent of the actual mouse embryo from E5.0 to 6.5 (Harrison et al., 2017). They recapitulate epiblast and trophoblast lumenogenesis, pro-amniotic cavity formation as well as mesoderm and primordial germ cell (PGC) induction.

Whereas in gastruloids the developing Bra/T pole or posterior seems to have no directional preference in the spherical aggregate, in ETS embryos, on the other hand, the presence of TSCs spatially confines the emergence of the this Bra/T domain at the boundary between embryonic and extraembryonic compartments, akin to the natural embryo. This role of the extraembryonic tissues is in congruence with the in vivo situation, where they provide not only the right signaling environment but also the crucial geometric cues that confer robustness to a developing embryo (Arnold and Robertson, 2009; Langdon and Mullins, 2011; Christodoulou et al., 2019).

This system was further complemented by adding a third cell type, XEN cells, which augment morphological similarity to in vivo mouse embryos by adding a visceral endoderm (VE)-like layer (Sozen et al., 2018; Zhang et al., 2019). Moreover, resulting ETX embryos display EMT, and subsequently meso- as well as endodermal marker expression, thus mimicking mouse embryo shape and morphogenesis until E7.0–7.5. It must be noted that, while such ETX embryos could initiate implantation responses in mouse uteri upon transplantation, they do not progress beyond this stage at the present time.

Replacing XEN cells with induced endodermal cells (transgenic mESCs featuring transient Gata4 upregulation) yields inducible ETX (iETX) embryos which more faithfully recapitulate particularly anterior morphogenetic events during mouse gastrulation (Amadei et al., 2021). This appears to be due to the previously employed XEN cells resembling the parietal endoderm rather than primitive endoderm, whereas induced endodermal cells exhibit increased developmental potential. iETX embryos hence form an anterior signaling center and display anterior visceral endoderm (AVE) migration, two hallmarks of mouse gastrulation.

### Mouse and human - blastoids

Blastoids, originally made from aggregated mESCs and TSCs, illustrate how the two cell types influence each other to form structures closely resembling mouse E3.5 blastocysts (morphologically and transcriptionally) with an outer trophoectoderm layer encapsulating the blastocoel, a fluid filled cavity, as well as the epiblast (Rivron et al., 2018). It appears that, not only do the TSCs guide the ESCs along their native developmental trajectory, the ESCs in turn maintain proliferation and self-renewal of TSCs as well as the trophoblast epithelial morphogenesis.

Although blastoids will not form bona fide embryos, they are capable of eliciting an implantation-like response in vivo, thereby constituting a potentially useful model system for studying mouse pre-gastrulation patterning dynamics. Other works also demonstrate the assembly of similar systems (Sozen et al., 2019; Kime et al., 2019).

In particular, EPS-blastoids are generated from extended pluripotent stem cells (EPSCs), pluripotent SCs cultured under conditions which enable both embryonic and extraembryonic developmental potential, as well as TSCs. These generate a primitive endoderm (PE)-like layer which, unlike normal blastoids, gives rise to cells expressing parietal endoderm markers. Moreover, under specific culture conditions, a subset of EPS-blastoids proceed to develop a post-implantation embryo-like morphology, similar to ETX embryos and E5.0-E5.5 cultured mouse embryos. The majority of these advanced EPS-blastoids also form an outer visceral endoderm (VE)-like layer.

A series of very recent studies with hPSCs (induced or otherwise) demonstrated that also human blastocyst-like structures (hBlastoids) can be successfully obtained by either mixing hESCs with SCs from extra-embryonic lineages or via employing naïve hEPSCs, akin to the mouse model (Liu et al., 2021; Yu et al., 2021; Sozen et al., 2021; Fan et al., 2021).

### Mouse and human - gastruloids

By expanding on culture conditions of mGastruloids, further research efforts have coaxed these into recapitulating additional aspects of native embryo morphology. For example, a recent work reports the application of matrigel to generate somite-like structutures in conjunction with respective gene oscillation during mGastruloid elongation (van den Brink et al., 2020). A parallel study adds that Wnt inhibition in conjunction with matrigel embedding further promotes anterior segment formation and improves physical separation of somites (Veenvliet et al., 2020).

Upon administration of cardiogenic factors FGF, VEGF and ascorbic acid, mGastruloids reproducibly recapitulate cardiogenesis at their anterior end, giving rise a vascular-like network, first and second heart fields as well as ultimately a beating structure, resembling an actual embryonic heart (Rossi et al., 2021). Moreover, mGastruloids generated not only from mESCs but also from XEN cells (‘neuruloids’) exhibit increased cell type diversity and develop neural-tube-like structures (Berenger-Currias et al., 2020).

Another study details the generation of post-implantation epiblast-like (EPI) gastruloids which, in contrast to standard mGastruloids, specify anterior neural tissues upon additional Wnt inhibition via XAV939, while still developing an elongated morphology (Girgin et al., 2021b). This was achieved by aggregating mESCs in an epiblast identity inducing medium with added Fgf2 and Activin-A, followed by continued exposure for 72 hr.

Epi-aggregates were combined with TSC aggregates to generate structures labeled EpiTS embryoids that undergo axial elongation and concomitant patterning (Girgin et al., 2021a). Addition of matrigel to epithelialize the Epi-aggregates restricts the onset of Bra/T expression at the boundary between Epi-ESCs and TSCs, akin to the native embryo as well as the ETS/ETX embryo. At later stages (anterior) neural patterning is augmented whereas mesendoderm differentiation is diminished.

In a conceptually similar approach, two disparately sized ESC aggregates were fused after an initial aggregation and growth phase (Xu et al., 2021), with the smaller one serving as a signaling center through brief exposure to BMP4, a factor known to induce gastrulation and axis formation in mammals (Winnier et al., 1995). Resulting embryo-like structures specify the three germ layers and proceed to develop until the equivalent of mouse embryo E9.0. They further exhibit advanced morphogenesis compared to standard mGastruloids, with, for instance, neural plate and tube-like structures, although these still appear to lack substantial forebrain and anterior midbrain populations.

Regarding human systems, it was recently demonstrated that pretreatment of human iPSCs with a Wnt agonist and FGF2 for 48 hr, followed by aggregation and maintenance in shaking culture, supplemented for 4 days with a growth factor cocktail (FGF2, HGF and IGF-1) yields gastruloids, termed EMLO, that can be grown over several weeks (Olmsted and Paluh, 2021). Overall, these appear to be biased toward trunk mesendoderm and central as well as peripheral nervous system fates. In particular, EMLO gastruloids display more complex morphology and neural cell type diversity when compared to standard hGastruloids, developing a primitive gut tube-like structure and, notably, innervation thereof.

### Human - amniotic-sac-like embryoids

By employing a microfluidic device, researchers were able to recapitulate post-implantation embryo development prior to and during early gastrulation, encompassing epiblast luminogenesis, formation of the bipolar embryonic sac and PGC as well as primitive streak cell specification (Zheng et al., 2019). To achieve this, single hESCs were grown on gel pockets within a customized three channel device, enabling cell loading and consecutive medium switching.

Following lumenogenesis, dorsal amniotic ectoderm-like cells were induced via localized application of BMP4. Ventral epiblast-like cells give rise to subpopulations expressing primitive streak markers such as BRA/T and PGC-associated genes. After 2–3 days in culture, cysts collapse due to emigration of ventral cells.

Altogether, the above examples illustrate, in systems from both mouse and human, how embryo-like morphology can be recapitulated through mimicking of an extraembryonic environment or associated cues which provide morphogenetic instructions.

### Outlook

In conjunction with the observed diversity in (pre-) gastrulation embryo shapes, comparison of in vitro and ex vivo systems to their in vivo counterparts may point toward an underlying developmental morphospace spanning a plethora of available trajectories (Mitteroecker and Huttegger, 2009).

**Figure 3.**
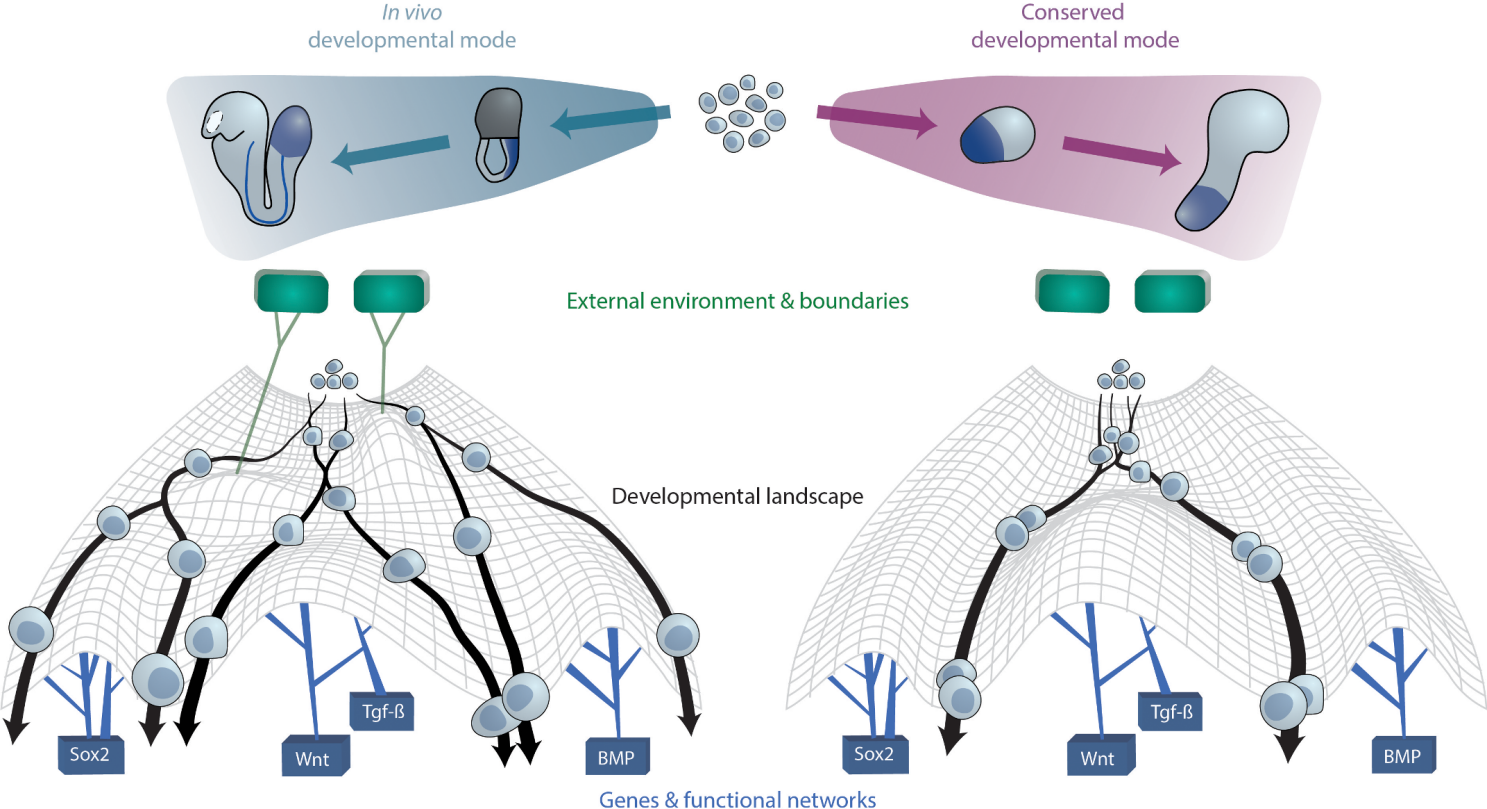
A conserved developmental mode emerges upon removal of species-specific extraembryonic environments. The developmental trajectories which aggregates of ESC(-LP)s exhibit upon removal of external or extraembryonic and associated boundaries may constitute a conserved mode that is shared across species. On a cellular level, this can be visually approximated as cells undergoing differentiation within Waddington’s developmental landscape Waddington, 1957. The landscape is shaped by key gene networks which remain constant between species and in vitro (bottom) as well as the external (micro-)environment and embryo geometry (top), here represented as green tiles, which vary between species. In case the latter factors are not present as ESCs are removed from their native context and grown in vitro, cellular developmental trajectories revert to the aforementioned conserved mode since cells from different species now experience the same landscape.

Since embryos of a given species generally develop under the same specific inputs, including geometry and extraembryonic environment, ESCs are developmentally biased and such alternative trajectories are revealed only when cells are removed from their native context and challenged by different external conditions (Figure 3). Furthermore, as illustrated above, ESC populations appear to possess an inherent capability to at least form a primary axis through a conserved developmental mode, which surfaces when ESC(-LP)s are grown in the most minimal conditions.

### A conserved developmental mode versus the influence of species-specific extraembryonic environments

When comparing systems such as gastruloids with blastoids and ETS/ETX embryos, it becomes apparent that, while the latter (mostly) faithfully recapitulate embryo geometry and morphogenesis, they are, at the present time, not able to develop beyond the in vivo equivalent of embryonic stage E7.0-E7.5. On the other hand, gastruloids do not look like actual embryos and undergo a more abstracted morphogenesis, yet they are capable of reaching the partial (transcriptional) equivalent of E9.0 (in case of the mESC-based system).

These observations raise an interesting point: It seems that by employing additional extraembryonic cell types or, in general terms, by mimicking a set of in vivo cues, resulting embryo-shaped artificial systems remain more constrained in their developmental potential. This is likely because, once having assumed an embryo-like developmental mode early during their respective morphogenesis, such structures would require further precise, spatially and temporally allocated inputs, just like the in vivo counterpart, in order to facilitate successive patterning events. In this context, it has to be mentioned that with advances in biomaterials and biotechnology, it should be possible to mimic extraembryonic environments more closely in the future (Gritti et al., 2021).

Gastruloid-like aggregates from pure ESCs or ESC-LPs, on the other hand, might represent a more unconstrained system, which is what enables them to develop beyond the equivalent of initial gastrulation, and therefore unveil the extent of inherent self-organizational capabilities of the source cells.

In summary, the wide range of trajectories displayed by ESCs and ESC-LPs in vitro converge onto an aforementioned conserved developmental mode under minimal conditions and collapse partly onto their native trajectories (at least for the mammalian systems) when provided with external inputs to recapitulate species-specific external environments (mechanical and chemical), facilitating increased morphological resemblance to the native embryo.

Characterizing this fundamental mode may perhaps ultimately provide insights into the origin and evolution of animal body plans as well as how the complexity of animal shapes during peri-gastrulation development may have arisen via distinct extraembryonic factors. In order to properly address this from a comprehensive evolutionarily perspective, however, more phylum coverage is required, dictating the need for novel minimal in vitro embryo-like systems from, for instance, diploblasts and protostomes.

### Evolution of early embryo morphology diversification

Concerning the significance of extraembryonic environments, it could be argued that, from the multitude of ways that pre-gastrulating embryos have at their disposal to establish their initial body plan, refinement of a species-specific developmental mode as well as corresponding (extra-)embryonic morphology over the course of evolution ensures robustness (or canalization) (Siegal and Bergman, 2002; Félix and Wagner, 2008; Wagner, 2008; Melzer and Theißen, 2016): Those developmental trajectories that worked most robustly in the specific ecological and reproductive niches were further specified to maximize fitness (Kalinka and Tomancak, 2012).

While this hypothesis remains unverified in metazoan embryos at large, a recent study finds that diversification of insect egg size and shape, traits which vary greatly across species, are driven by shifts in the respective oviposition microenvironments, that is, where the eggs are laid (Church et al., 2019). Likewise, it was functionally demonstrated that coordinated adherence of the insect blastoderm to the vitelline envelope, a structure or shell surrounding the developing embryo, influences tissue movement dynamics during gastrulation (Münster et al., 2019), further highlighting the importance of morphology and embryonic-extraembryonic interactions in early patterning processes.

Such external or extraembryonic environments and their complexity vary greatly across species and constitute a substantial challenge to replicate in vitro. It is natural to speculate that this could be one of the reasons why it is more straightforward to generate a functional *Nematostella* or Starfish larva as opposed to a fish, mouse, or human from isolated ESC-like cells.

Nonetheless, comparing these different kinds of embryo-like structures should therefore provide tangible clues as to which aspects of embryonic development or, more precisely, axis formation and associated morphogenesis are inherent to ESCs and which parts strictly require extraembryonic input.

### The phylotypic period and further developmental modes

We note that there are established concepts related to conservation of body plans and gene expression across species: The developmental *hourglass* model delineates a phylotypic period at mid-embryogenesis during which common anatomical features (the basic body plan) for each respective phylum are established (Duboule, 1994; Raff, 1996). In turn, this period is developmentally preceded and followed by phases of increased morphological and transcriptional divergence, thereby shaping a developmental ‘hourglass’ (Kalinka and Tomancak, 2012; Kalinka et al., 2010; Irie and Kuratani, 2014).

A related term is ‘mid-developmental transition’ (MDT), referring to a transition period between early and late stages of conserved gene expression which is characterized by phylum-specific activity of gene networks (Kalinka et al., 2010). This phase, overlapping with the phylotypic period in previously studied animals, has been identified from sponges to chordates and may therefore serve to define a phylum as a group of species that, during the MDT, exhibit gene expression which is convergent among themselves but divergent to other species (Levin et al., 2016).

The conserved developmental mode that we propose here, on the other hand, applies to ESCs across phyla (albeit we acknowledge that this inference may be erroneous due to limited taxon coverage of existing minimal in vitro systems). Moreover, this mode entails the establishment of primary axial identity from an initially homogeneous or disorganized cell population, whereas the MDT and phylotypic period span the organogenesis phase of embryonic development, at which point the respective body axes are already specified.

However, it can be conjectured that future study of embryo-like systems may reveal further developmental modes, associated with the emergence of corresponding dynamical patterning modules(Newman and Bhat, 2008; Newman, 2016). It is likely that these would not be universally conserved across the animal kingdom, but rather among phyla or lower taxonomic ranks, given that events following AP or OA axis specification often generate increasingly phylum or species-specific features of the larval or adult body plan. Additional developmental modes could thus surface during the organogenesis stage of development and pertain to specific organ (primordia) specification or morphogenesis.

Taken together, establishment of new in vitro embryo-like systems in conjunction with refinement of existing ones and investigation of their displayed initial patterning dynamics will help to assess if or to which degree aforementioned conserved developmental modes exist. Hence, integration and comparison of such studies with data from actual embryos should aid to more concisely delineate underlying mechanisms of primary axis and subsequent initial body plan specification in cell ensembles as well as to elucidate early (pre- and peri-gastrulation) embryo morphology diversification during evolution.
